# Matrine reverses the resistance of *Haemophilus parasuis* to cefaclor by inhibiting the mutations in penicillin-binding protein genes (*ftsI* and *mrcA*)

**DOI:** 10.3389/fmicb.2024.1364339

**Published:** 2024-03-15

**Authors:** JingChao Zhao, Wen Yang, Hui Deng, Dong Li, QianYong Wang, LingXian Yi, QiHong Kuang, Rui Xu, Di Li, RuoNan Li, DaoJin Yu, Bo Yang

**Affiliations:** ^1^University Key Laboratory for Integrated Chinese Traditional and Western Veterinary Medicine and Animal Healthcare in Fujian Province, Fujian Agriculture and Forestry University, Fuzhou, Fujian Province, China; ^2^Fujian Key Laboratory of Traditional Chinese Veterinary Medicine and Animal Health, Fujian Agriculture and Forestry University, Fuzhou, Fujian Province, China; ^3^Wuhan Animal Disease Control Center, Wuhan, Hubei Province, China

**Keywords:** *Haemophilus parasuis*, matrine, cefaclor, resistance, penicillin-binding protein, *ftsI*, *mrcA*

## Abstract

**Introduction:**

Matrine (MT) is a potential resistance reversal agent. However, it remains unclear whether MT can reverse the resistance of *Haemophilus parasuis* (*H. parasuis*) to β-lactams, and, if so, by what mechanism MT works.

**Methods:**

We screened one cefaclor (CEC)-resistant strain (clinical strain C7) from eight clinical (*H. parasuis*) strains and determined the underlying resistance mechanism. Then, we investigated the reversal effect of MTon the resistance of this strain to CEC.

**Results and Discussion:**

The production of β-lactamase, overexpression of AcrAB-TolC system, and formation of biofilm might not be responsible for the resistance of clinical strain C7 to CEC. Fourteen mutation sites were found in four PBP genes (*ftsI, pbp1B, mrcA*, and *prcS*) of clinical strain C7, among which the mutation sites located in *ftsI* (Y_103_D and L_517_R) and *mrcA* (A_639_V) genes triggered the resistance to CEC. The minimum inhibitory concentration (MIC) of CEC against clinical strain C7 was reduced by two to eight folds after MT treatment, accompanied by the significant down-regulated expression of mutated *ftsI* and *mrcA* genes. Based on such results, we believed that MT could reverse the resistance of *H. parasuis* to CEC by inhibiting the mutations in *ftsI* and *mrcA* genes. Our research would provide useful information for restoring the antimicrobial activity of β-lactams and improving the therapeutic efficacy of Glässer’s disease.

## Introduction

Glässer’s disease is a bacterial infectious disease caused by *Haemophilus parasuis* (*H. parasuis*). It is usually clinically manifested as serositis, arthritis, bronchitis, meningitis, and septicemia, and has caused huge economic losses to the pig industry (Costa-Hurtado et al., 2020). β-lactams may be the current drugs of choice for the treatment of Glässer’s disease because of their powerful antibacterial activity and good safety. However, the surveys on the prevalence and antimicrobial susceptibilities of *H. parasuis* from 2013 to 2017 in China revealed high resistance rates and increasing resistance to β-lactams (Zhao et al., 2018; Zhang et al., 2019), which posed a serious challenge to the effectiveness of β-lactams in the treatment of Glässer’s disease. Thus, there is an urgent need to explore new and more effective therapies.

Screening of resistance-reversal agents from phytochemicals and combining them with antimicrobials is expected to address the challenge of antimicrobial resistance (Ayaz et al., 2019; Khameneh et al., 2019). Some phytochemicals show little or no antimicrobial activity. Nonetheless, they can neutralize the resistance mechanism, enabling the antimicrobials to still be effective against resistant microbes (Zhou et al., 2012; Cai et al., 2016; Barbieri et al., 2017; Pourahmad and Mohammadi, 2018; Itzia Azucena et al., 2019; Qu et al., 2019; Wei et al., 2020). Matrine (MT) is one of the main quinolizidine alkaloids extracted from plants such as *Sophora flavescens*, *Sophora alopecuroide*, and *Sophora subprostrata* (Pourahmad and Mohammadi, 2018). Previous *in vitro* experiments showed that MT reversed the resistance of *Escherichia coli* (*E. coli*) to a variety of antimicrobials by inhibiting the AcrAB-TolC efflux pump (Zhou et al., 2012; Pourahmad and Mohammadi, 2018). Another study revealed the inhibitory effect of MT on the biofilm formation of antimicrobial-resistant *E. coli*, which helped to reduce the resistance to antimicrobials from the aspect of interbacterial population communication (Sun et al., 2019). In addition to the predictable resistance reversal activity, MT also exhibits good pharmacokinetic properties and safety (You et al., 2020), which makes it a promising resistance-reversal agent for veterinary clinical use. However, it remains unclear whether MT can also reverse the resistance of *H. parasuis* to β-lactams, and, if so, by what mechanism MT works.

The objectives of this study were to investigate the reversal effect of MT on the resistance of *H. parasuis* strains to β-lactam*s* and the underlying mechanism. The results would provide a theoretical basis for the further application of MT in veterinary practice.

## Materials and methods

### Chemicals

The standards of MT, ampicillin (AMP), amoxicillin (AMX), oxacillin (OXA), penicillin (PEN), ceftiofur (CEF), cefaclor (CEC), cefepime (CPE) and cefotaxime (CTX) were purchased from Shanghai Yuanye Bio-Technology Co. Ltd. (Shanghai, China) with a purity ≥95%. Stock standard solutions of MT and 8 β-lactams were prepared separately by dissolving each compound in sterile distilled water at a concentration of 5,120 μg/mL and then stored at −20°C. Carbonyl cyanide 3-chlorophenylhydrazone (CCCP), as an efflux pump inhibitor, was purchased from Macklin Inc. (Shanghai, China) with a purity ≥98%. Stock standard solution of CCCP was prepared in dimethylsulfoxide (DMSO) at a concentration of 200 mg/mL and then stored at −20°C. Other chemical reagents were of analytical grade and purchased from the Sinopharm Chemical Reagent (Shanghai, China).

### Bacterial strains and culture conditions

A total of 8 clinical strains of *H. parasuis* were used in this study. Six strains were kindly provided by National Reference Laboratory of Veterinary Drug Residues at Huazhong Agricultural University (Wuhan, China). The other two strains were obtained from Fujian Academy of Agricultural Sciences (Fuzhou, China). These strains were isolated from the diseased pigs suffering fibrinous polyserositis, polyarthritis, and meningitis diagnosed in China during March 2014 to May 2018, and were confirmed by polymerase chain reaction (PCR). All of the clinical strains were stored at −80°C in milk. Prior to use, each strain was sub-cultured at least three times on modified tryptone soya agar (TSA) (BD Biosciences, Franklin Lakes, NJ, United States) supplemented with 5 μg/mL of nicotinamide adenine dinucleotide (NAD) (Sigma-Aldrich, St. Louis, MO, United States) and 5% (v/v) new-born calf serum (Tianhang Biotechnology Co. Ltd., Huzhou, Zhejiang Province, China) to ensure viability and purity. Tryptone soya broth (TSB) (BD Biosciences, Franklin Lakes, NJ, United States) and modified TSB supplemented with 5 μg/mL of NAD and 5% (v/v) new-born calf serum were used for screening of β-lactam-resistant strains, determining possible resistance mechanism, and investigating the reversal effect of MT on the resistance of *H. parasuis* strains to β-lactams. *E. coli* (ATCC25922) and *Staphylococcus aureus* (*S. aureus*, ATCC25923 and ATCC29213) were kept in our laboratory.

### Screening of β-lactam-resistant strains

The susceptibility of 8 clinical *H. parasuis* strains to β-lactams was determined using the microdilution method according to the Clinical and Laboratory Standards Institute (CLSI, 2020). Eight β-lactams were tested, included AMX, AMP, OXA, PEN, CEC, CTX, CEF, and CPE. Aliquots of modified TSB supplemented with β-lactams (100 μL) at concentrations ranging from 0.015625 to 256 μg/mL were prepared in a 96-well plate. An exponentially growing bacterial culture was diluted with modified TSB to 1.0 × 10^6^ cfu/mL, and then 100 μL of diluted bacteria was added to each treatment. The mixture was incubated at 37°C for 24–48 h. *E. coli* (ATCC25922) and *S. aureus* (ATCC29213) were used for quality control. All experiments were repeated three times. The minimum inhibitory concentration (MIC) was defined as the lowest concentration at which the wells remained clear. β-lactam-resistant strain was identified according to the determined MICs and the breakpoints established by Chen et al. (2021). The MIC of MT against β-lactam-resistant strain was also determined as described above.

### Determination of possible resistance mechanism

β-lactamase activity in β-lactam-resistant strain was assessed by nitrocefin test and enzyme linked immunosorbent assay (ELISA). The tested strain was sub-cultured twice, then a single colony was selected and applied to nitrocefin paper (Wenzhou Kangtai Biotechnology Co. Ltd., Wenzhou, China). Visual observation of the colour change of the nitrocefin paper was performed after standing at room temperature for 5 min. *S. aureus* (ATCC29213) and *S. aureus* (ATCC25923) were used as negative and positive controls, respective. ELISA was performed according to the manufacturer’s instructions of β-lactamase ELISA kit (Shanghai Zhuochai Biology Co. Ltd., Shanghai, China). All experiments were repeated three times. β-lactamase-producing strain was identified according to the colour change (from yellow to red) of the nitrocefin paper and the results of ELISA.

Multidrug efflux pump activity in β-lactam-resistant strain was assessed by comparison the MICs to β-lactams before and after treatment with CCCP. For the treatment group, each test well contained CCCP at a final concentration of 10 μg/mL. The MICs were determined as described above. Efflux pump-positive phenotype was identified when the ratio of MIC to MIC__CCCP treatment_ was equal to or greater than 4.

Biofilm-forming ability of β-lactam-resistant strain was assessed by crystal violet staining. Briefly, 10 μL of exponentially growing bacterial culture (1.0 × 10^8^ cfu/mL) was added into individual well of a 96-well plate containing 100 μL of modified TSB. The well containing only modified TSB was used as control. Then, the 96-well plate was incubated at 37°C for 48 h. After incubation, the plate was washed three times with 200 μL of PBS (pH = 7.2) and fixed with 100 μL of methanol for 15 min. The excess methanol was discarded and the plate was left to dry at ambient temperature. The plate was then strained with 1% (w/v) crystal violet for 5 min. Unbound dye was wash away with PBS (pH = 7.2) and the plate was air-dried again. Finally, 100 μL of 33% (v/v) glacial acetic acid was added to each well and the optical density (OD) was measured at 630 nm using a microplate reader (Bio-Rad Laboratories, Hercules, CA, United States). All experiments were repeated three times. Strain which has strong ability to form biofilm was identified when the ratio of OD__test_ to OD__control_ was equal to or greater than 2.

The presence of antimicrobial-resistant genes (ARGs) was determined by PCR in β-lactam-resistant strain. These ARGs included *bla_TEM_*, *bla_OXA_*, *bla_SHV_*, *bla_CTX_*, *bla_IMP_*, *bla_DHA_*, *bla_ROB_*, *bla_ROB1_*, and *acrA*. The genomic DNA was extracted using a TIANamp Bacteria DNA Kit (Tiangen Biotech Co. Ltd., Shanghai, China), according to the manufacture’s instruction. The primers for PCR (Table 1) were synthesized by Sangon Biotech (Shanghai) Co. Ltd. (Shanghai, China). The PCR system contained 12.5 μL of 2 × CataAmp TaqPCR Mix (Beijing Catascis Biotech Co. Ltd., Beijing, China), 2 μL of DNA template, 1 μL of forward primer, 1 μL of reverse primer, and 8.5 μL of RNase-free water. Amplification conditions began with an initial denaturation at 98°C for 3 min, followed by 30 cycles of 98°C for 10 s, 52–59°C for 20 s, and 72°C for 20 s, and terminated at 72°C for 10 min.

**Table 1:** PCR primers used in this study.

Gene	Primer sequence (5′–3′)	Annealing temp (°C)	Produce size (bp)	Reference
*bla*_TEM_	GAGTATTCAACATTTTCGT ACCAATGCTTAATCAGTGA	55	856	Dayao et al. (2016)
*bla_OXA_*	GCAGCGCCAGTGCATCAAC CCGCATCAAATGCCATAAGTG	58	198	Liu (2013)
*Bla_CTX_*	GGCGTTGCGCTGATTAACAC TTGCCCTTAAGCCACGTCAC	59	486	Liu (2013)
*Bla_SHV_*	TCGCCTGTGTATTATCTCCC CGCAGATAAATCACCACAATG	56	768	Liu (2013)
*bla_IMP_*	TGAGGCTTACCTAATTGACA TCAGGCAACCAAACCACTAC	56	324	Liu (2013)
*bla_DHA_*	ATCTGCAACACTGATTTCCG TTACACTAGGGGAAGGTGACG	56	360	Liu (2013)
*bla_ROB1_*	CATTAACGGCTTGTTCGC CTTGCTTTGCTGCATCTTC	55	852	Dayao et al. (2016)
*acrA*	GAAGTGATTATCCGTACTTG CAATAGGCTAATACAGATAG	55	1,337	This study
*ftsI*	ACCGCTTGCACTATATAAGACTAAC TAGTGCGTGTGGTGGTGGCTGA	58	2,130	Liu (2013)
*ftsI2*	AAAGTCAGAAGTTTTTAGTCGGG GGCTGTCCAAGGTCATTTTG	57	2,246	Liu (2013)
*mrcA*	ATAAGCAGGGGGCAGTTTTC TACCACAAATCGCCCAAAATAA	58	2,725	Liu (2013)
*pbp1B*	GGGCAATAATAGAGAAATATACGTG TACGCAGAGTTGAACGCAAAGT	60	2,607	Liu (2013)
*dacA*	AATATGAGAGCGGTGTGATTTTGCA TGAAGAAGCTCAACACGTTAAACAG	60	1,366	Liu (2013)
*dacB*	ACTGTTGTTACAGGTTTAAGTCGGT AGAGAATGTCTTGGCAGAAAAAGAG	60	1,038	Liu (2013)
*prcS*	AGAAAGTTTGTCCGCATTAGA ACCCCTTTAATTTGGGTGCT	59	1,630	Liu (2013)
*prcA*	AAATCAGACCGCTTAAACAGC TTAACCGTCATAGTGCTTCG	60	722	Liu (2013)

The mutation of penicillin-binding proteins (PBPs) genes in β-lactam-resistant strain was detected by PCR amplification and conventional DNA sequencing. These PBP genes included *ftsI*, *ftsI2*, *mrcA*, *pbp1B*, *dacA*, *dacB*, *prcA*, and *prcS*. The primes for PCR (Table 1) were synthesized by Sangon Biotech (Shanghai) Co. Ltd. (Shanghai, China). The PCR system contained 25 μL of 2 × CataAmp High Fidelity PCR Mix (Fuzhou Yihe Gene Technology Co. Ltd., Fuzhou, China), 1 μL of DNA template, 2 μL of forward primer, 2 μL of reverse primer, and 20 μL of RNase-free water. The DNA extraction and PCR amplification were performed as described above. The amplified products were sequenced by Sangon Biotech (Shanghai) Co. Ltd. (Shanghai, China). The mutations in PBP genes were identified by comparing the sequencing results with the corresponding sequences in *H. parasuis* SH0165. To identify the association between the mutations in PBP genes and the resistance to β-lactams, the mutated PBP genes were cloned into PLB-T vector (Tiangen Biotech Co. Ltd., Beijing, China), and transformed into *E. coil* DH5α (Tiangen Biotech Co. Ltd., Beijing, China), according to the manufacturer’s instructions. Then, the susceptibility of these transformants to β-lactams was determined according to the microdilution method recommended by the CLSI (2020). Transformants containing wild-type PBP genes were used as controls.

### Investigation into the reversal effect of MT on the resistance to β-lactams

The changes in the susceptibility of β-lactam-resistant *H. parasuis* to CEC were determined after treatment with various concentrations of MT. Briefly, 100 μL of exponentially growing bacterial culture (1.0 × 10^8^ cfu/mL) was incubated with an equal volume of modified TSB contained various concentrations of MT (0, 0.25, 0.5, 4, 16, and 32 μg/mL) for 22 h at 37°C. Aliquots (100 μL) of the bacterial culture were taken at 1, 4, 7, 10, and 13 h and subsequently centrifugated at 3000 rpm for 5 min at 4°C. The pellets were washed with sterile PBS (pH = 7.2) to remove residual MT. Then, the bacteria were sub-cultured twice and cultured in modified TSB to the exponential growth phase (1.0 × 10^8^ cfu/mL). The MICs of CEC against the exponentially growing bacteria were determined as described above. All experiments were repeated three times.

The changes in the biofilm-forming ability of β-lactam-resistant *H. parasuis* were determined after treatment with various concentrations of MT (0, 0.125, 0.25, 2, 8, and 16 μg/mL). The incubation of β-lactam-resistant strain with MT and the assessment of biofilm formation were performed as described above. All experiments were repeated three times.

The morphological change of β-lactam-resistant *H. parasuis* was observed using scanning electron microscopy (SEM, Hitachi Higher Technologies Co., Tokyo, Japan) after treatment with MT at 16 μg/mL for 7 h. The incubation procedure was the same as described above.

The changes in the relative expression level of mutated PBP genes were determined by real-time fluorescence quantitative PCR (qRT-PCR), after the β-lactam-resistant *H. parasuis* was treated with various concentrations of MT. The incubation procedure was the same as described above. The total RNA was extracted using a RNAprep Pure Cell/Bacteria Kit (Tiangen Biotech Co. Ltd., Shanghai, China), according to the manufacture’s instruction. Then, the cDNA was synthesized according to the manufacture’s instruction of FastKing gDNA Dispelling RT Super-Mix kit (Tiangen Biotech Co. Ltd., Shanghai, China). QRT-PCR was performed with Taq SYBR Green qPCR Premix (Yeasen Biotechnology Co. Ltd., Shanghai, China) using the Roche LightCycler^®^ 384 RT-PCR system (Roche, Basel, Switzerland). Relative quantification was performed by the 2^−ΔΔCt^ method, and *ppiB* was used as internal control. The primers for qRT-PCR (Table 2) were synthesized by Sangon Biotech (Shanghai) Co. Ltd. (Shanghai, China). Each sample was measured with three technical replicates per target gene per independent experiment.

**Table 2:** qRT-PCR primers used in this study.

Gene	Primer sequence (5′–3′)	Annealing temp (°C)	Produce size (bp)	Reference
*ftsI*	CATCCAATCCGACATCTTATC TTCTACACAGCGTTTCCGT	60	138	This study
*mrcA*	GCGGTCGTAGTCAATCAAG TCCGTCAAGAAATGGTAAAAC	60	136	This study
*pbp1B*	GTCTGCTTATTGATGCGTTG TCTGCCTGCTTGATAGTTTG	60	112	This study
*ppiB*	ATCCTCATTCAGCCACCTCACA CAAAAACAGCATACCCCCATTC	60	120	This study

### Statistical analysis

All values were expressed as means ± standard deviation (SD). Statistical analysis was performed using two-tailed *t*-test with SPSS version 21 (IBM Co., Armonk, NY, United States). A *p*-value of <0.05 was considered statistically significant.

## Results

### Susceptibility of clinical *Haemophilus parasuis* strains to β-lactams

The MICs of eight β-lactams against eight clinical *H. parasuis* strains are shown in Table 3. Most strains were susceptive or intermediate to eight β-lactams. Only one strain (clinical strain C7) showed resistance to CEC. This strain was used for subsequent experiments. The MIC of MT against the clinical strain C7 was 512 μg/mL.

**Table 3:** The susceptibility of 8 clinical *H. parasuis* strains to 8 β-lactam antibiotics.

Strains No.	MIC (μg/ml)							
	AMX	AMP	OXA	PEN	CEC	CTX	CEF	CPE
C1	0.125(S)	0.0625(S)	1(S)	0.5(S)	1(S)	0.125(I)	0.5(I)	0.25(S)
C2	0.25 (S)	0.5(S)	2(S)	0.5(S)	0.25(S)	0.03125(S)	0.125(S)	0.25(S)
C3	0.125(S)	0.25(S)	1(S)	1(S)	0.5(S)	0.125(I)	0.25(S)	0.125(S)
C4	0.125(S)	0.0625(S)	1(S)	0.5(S)	0.125(S)	0.0625(S)	0.125(S)	0.125(S)
C5	0.0625(S)	0.5(S)	1(S)	0.25(S)	0.5(S)	0.0625(S)	0.25(S)	0.125(S)
C6	0.25(S)	0.0625(S)	0.5(S)	0.0625(S)	1(S)	0.125(I)	0.0625(S)	0.5(I)
C7	0.5(S)	0.25(S)	8(I)	0.125(S)	32(R)	0.125(I)	0.125(S)	0.5(I)
C8	0.125(S)	0.125(S)	4(S)	0.125(S)	8(S)	0.0625(S)	0.25 (S)	0.5(I)

S, sensitive; I, intermediate; R, resistant.

### Mechanism of resistance to CEC in clinical strain C7

**Figure 1:**
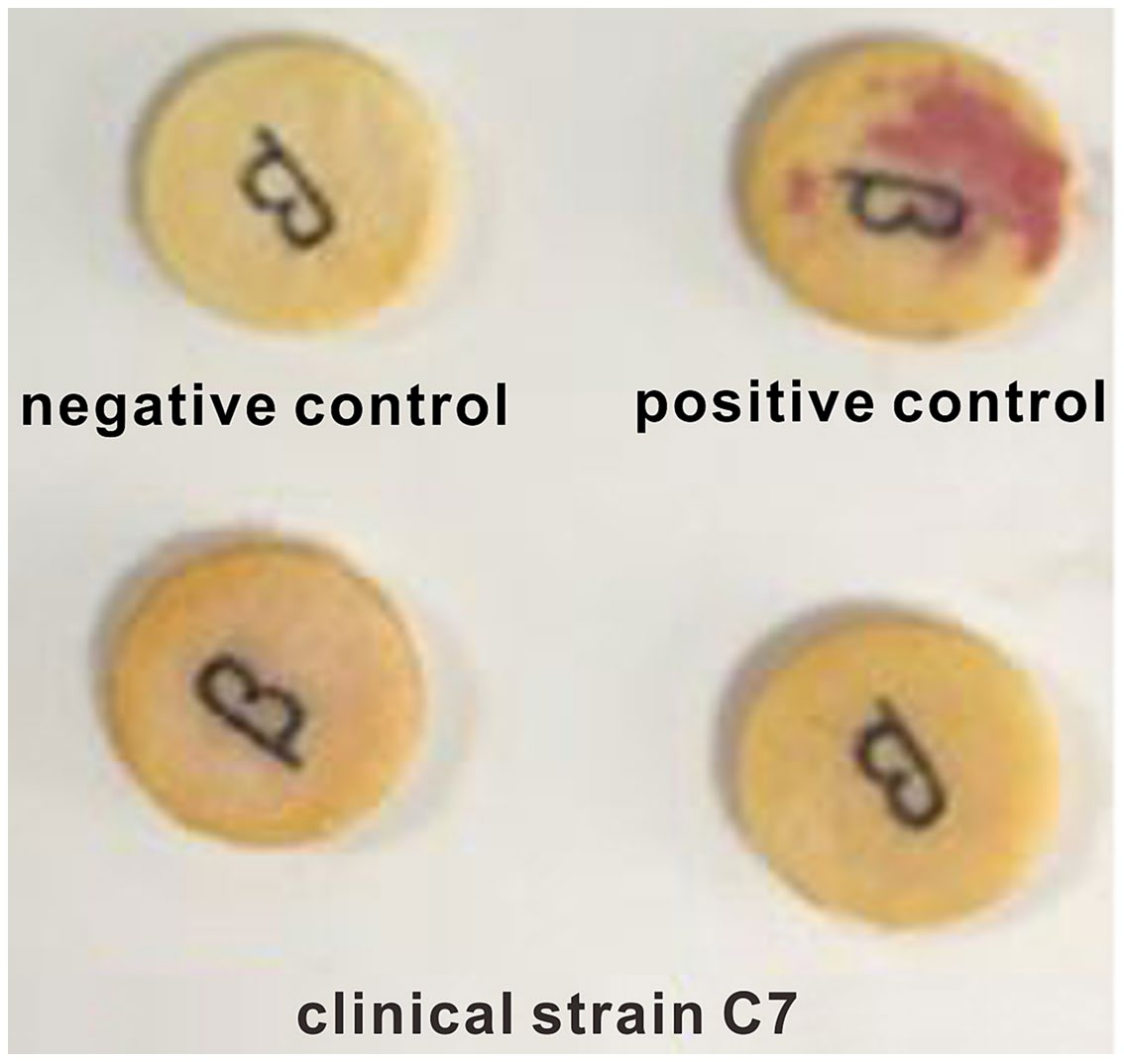
Representative results of nitrocefin test.

A negative result was observed in nitrocefin test (Figure 1). Similar result was obtained by ELISA assay. The β-lactamase content in clinical strain C7 was much lower than the limit of detection (0.1 U/L) of ELISA method. The consistency was also observed between phenotype and genotype. None of β-lactamase-encoding genes was found in clinical strain C7. These results indicated that the clinical strain C7 was not a β-lactamase-producing *H. parasuis*.

Efflux pump inhibition test showed that the ratio of MIC to MIC__CCCP treatment_ was equal to 2, which suggested the resistance of clinical strain C7 to CEC might not be associated with the overexpression of AcrAB-TolC efflux pump. This speculation was supported by the fact that *acrA* gene was not detected in clinical strain C7.

Crystal violet staining showed that the ratio of OD__test_ to OD__control_ was equal to 1.15, which suggested the clinical strain C7 had a weak ability to form biofilm.

**Figure 2:**
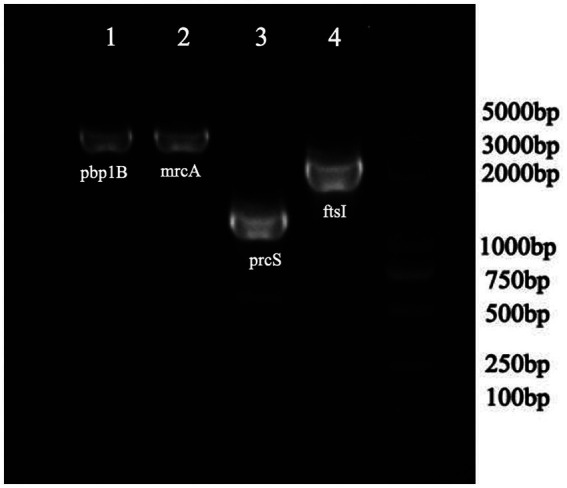
PCR analysis confirming the transformants containing the PLB-T vector with mutated PBP gens.

As shown in Table 4, a total of fourteen mutation sites were found in four PBP genes (*ftsI*, *pbp1B*, *mrcA*, and *prcS*). The presence of positive bands at 2607, 2725, 1,630, and 2,130 bp (Figure 2), coupled with subsequent sequencing results indicated that the transformants containing the PLB-T vector with mutated PBP genes had been successfully constructed. The MICs of transformants carrying mutated *ftsI* and *mrcA* genes to CEC were increased 4 and 2 times respectively, when compared with those of transformants carrying wild-type PBP genes. No changes in susceptibility to CEC were observed when the other mutated PBP genes (*pbp1B* and *prcS*) were cloned into PLB-T vector and then transformed into *E. coil* DH5α.

**Table 4:** Mutation sites of PBP genes in clinical strain C7.

PBP gene	Mutation site
*ftsI*	Y_103_D, L_517_R
*mrcA*	A_639_V
*pbp1B*	R_509_H, S_689_G, R_734_H, I_602_M
*prcS*	N_4_T, L_9_F, C_17_M, S_18_G, E_40_N, S_43_I, I_44_T

### Reversal effect of MT on the resistance to CEC in clinical strain C7

**Figure 3:**
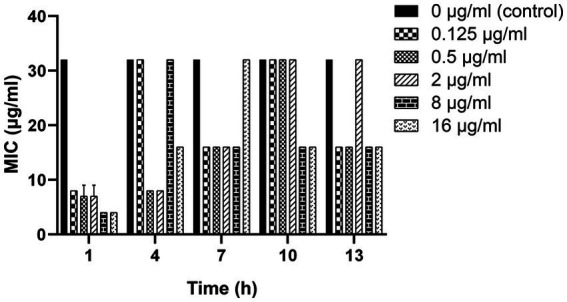
Changes in the susceptibility of clinical strain C7 to CEC after treatment with MT. Each bar represented the mean ± SD of the three independent experiments.

As shown in Figure 3, a 2-to 8-fold decrease in MIC was observed after the clinical strain C7 was treatment with various concentrations of MT for 13 h. However, such reversal effect did not appear to be concentration-or time-dependent.

**Figure 4:**
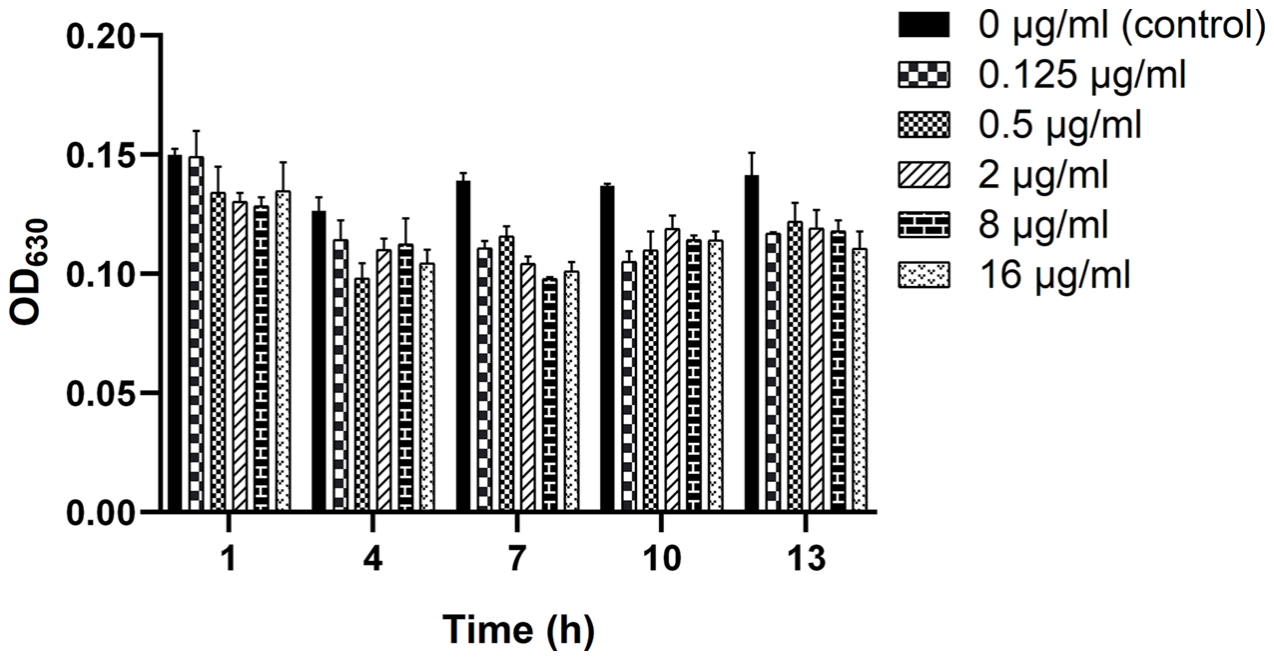
Changes in the biofilm-forming ability of clinical strain C7 after treatment with MT. Each bar represented the mean ± SD of the three independent experiments.

As shown in Figure 4, the biofilm-forming ability of clinical strain C7 was inhibited in the presence of low concentration of MT. However, such inhibitory effect was not significantly different (*p* > 0.05) from that of the control group (clinical strain C7 without treatment by MT).

**Figure 5:**
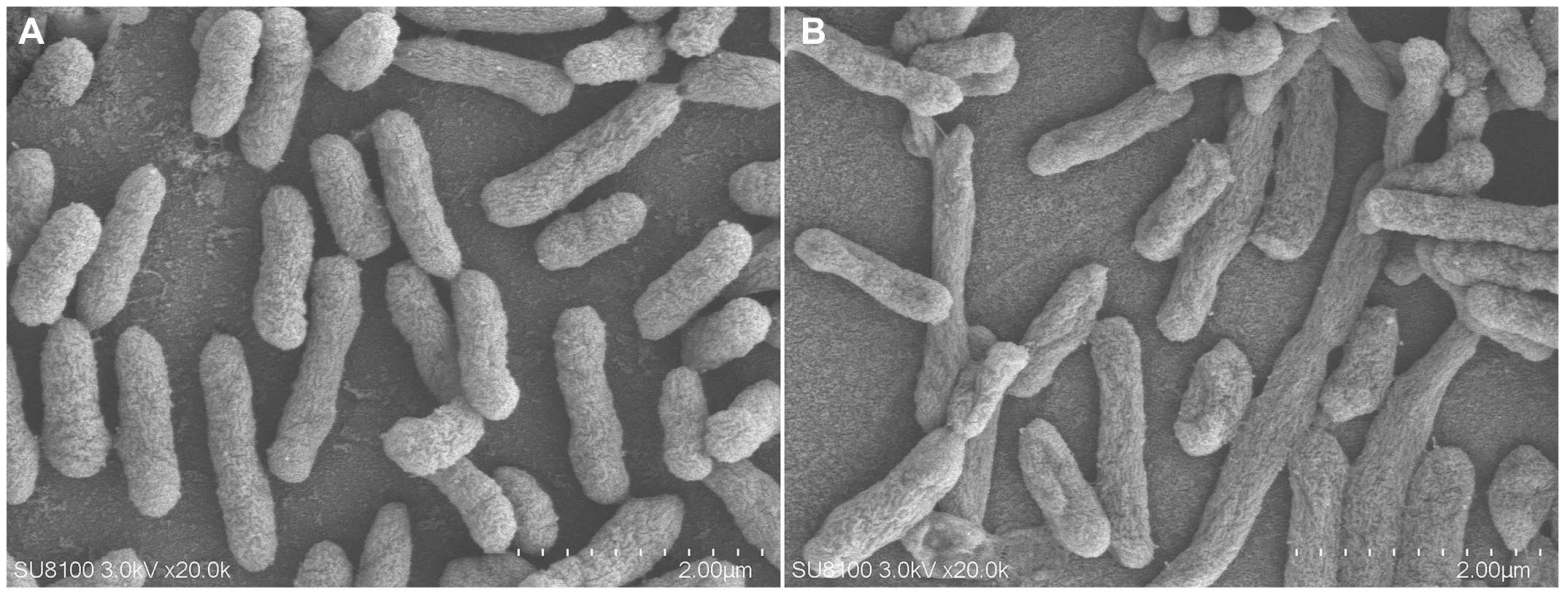
Morphological change of clinical strain C7 before **(A)** and after treatment with MT **(B)**.

As shown in Figure 5, compared with the control group, the bacterial membrane of clinical strain C7 became wrinkled and concaved after treatment with MT at 16 μg/mL for 7 h.

**Figure 6:**
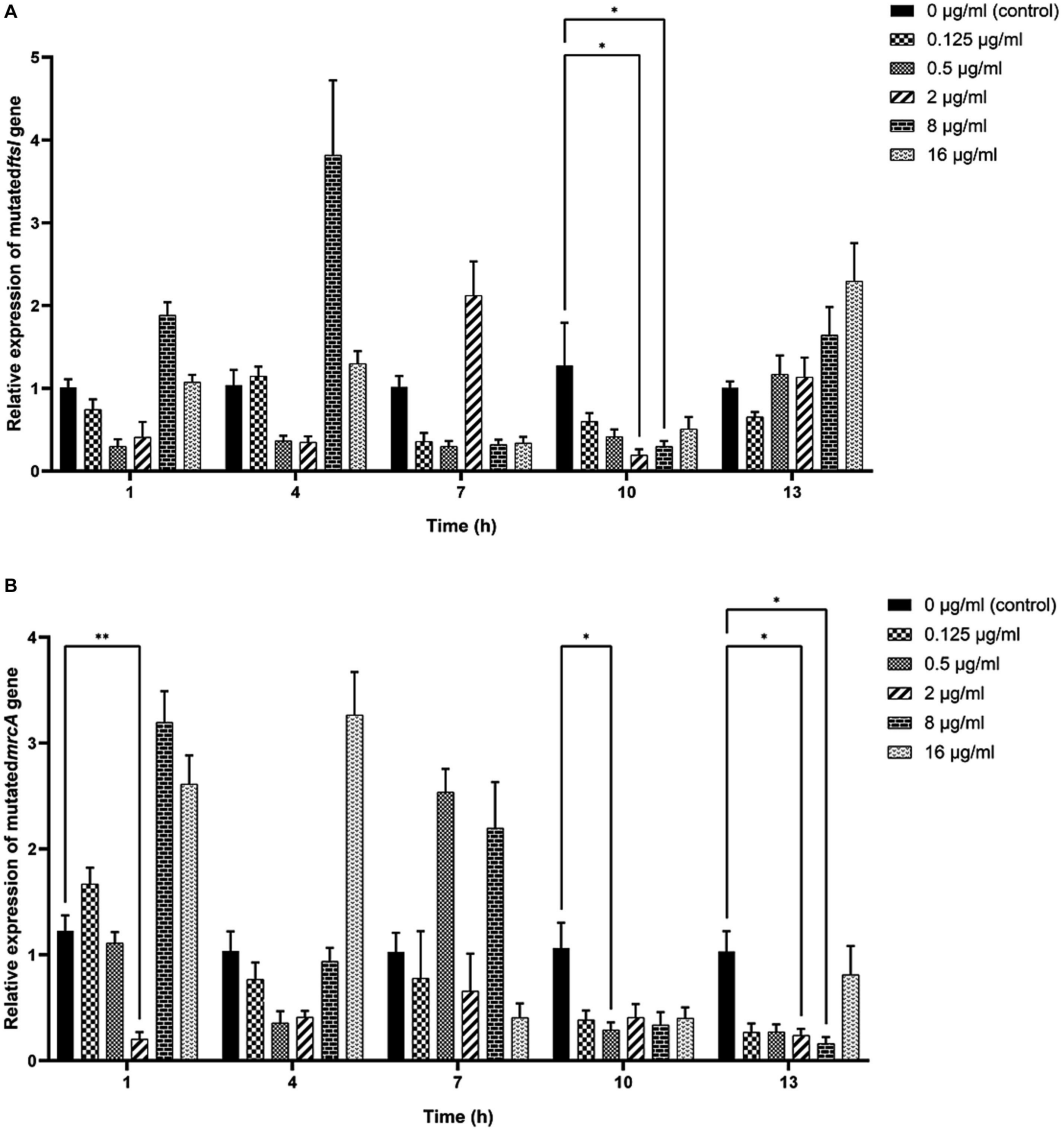
Effects of MT on the relative expression of mutated *ftsI* **(A)** and *mrcA* **(B)** genes after treatment with MT (^*^ indicated a significant difference at *p* < 0.05; ^**^ indicated a significant difference at *p* < 0.01). Each bar represented the mean ± SD of the three independent experiments.

As shown in Figure 6, significant decreases in the relative expressions of mutated *ftsI* and *mrcA* genes were observed after the clinical strain C7 was treated with various concentrations of MT for 1, 10 and 13 h. However, such changes did not appear to be concentration-or time-dependent too.

## Discussion

The widespread use of antimicrobials in livestock husbandry can induce antimicrobial resistance, which leads to the shortened service lifespan of antimicrobials and failure of anti-infective therapy. Screening of resistance-reversal agents and investigating their mechanism of action may be a promising strategy to address the challenge of antimicrobial resistance. In this study, we screened one CEC-resistant *H. parasuis* (clinical strain C7) from eight clinical *H. parasuis* strains and determined the underlying resistance mechanism. Then, we investigated the reversal effect of MT on the resistance of this strain to CEC. We believed that our research would provide useful information for restoring the antimicrobial activity of β-lactams and improving the therapeutic efficacy of Glässer’s disease.

Four possible mechanisms may contribute to β-lactam resistance in *H. parasuis*. First, the production of β-lactamase encoded by *bla_TEM_*, *bla_OXA_*, *bla_SHV_*, *bla_CTX_* and so no, which can inactivate β-lactams by hydrolyzing the β-lactam ring (San Millan et al., 2007; Yang et al., 2013; Moleres et al., 2015; An et al., 2023). Second, the overexpression of efflux pump such as AcrAB-TolC system, which can actively expel a wide range of toxic compounds, including β-lactams, from the cell (Feng et al., 2014). Third, the formation of biofilm, which was proved to be positive correlation with resistance to β-lactams (Zhang et al., 2014). Fourth, the mutations in PBP genes, which may reduce the affinity of PBP transpeptidases for β-lactams. To our knowledge, for *H. parasuis*, the association between the mutations in PBP genes and the resistance to β-lactams has not been identified. The results of our study showed that (i) the production of β-lactamase, overexpression of AcrAB-TolC system, and formation of biofilm might not be responsible for the resistance of clinical strain C7 to CEC; and (ii) fourteen mutation sites were found in four PBP genes (*ftsI*, *pbp1B*, *mrcA*, and *prcS*) of clinical strain C7, among which the mutation sites located in *ftsI* (Y_103_D and L_517_R) and *mrcA* (A_639_V) genes triggered the resistance to CEC, which was basically consistent with Liu’s prediction (Liu, 2013). This conclusion was also supported indirectly by the facts that the MIC of CEC against clinical strain C7 was reduced by two to eight folds after MT treatment, accompanied by the significant down-regulated expression of mutated *ftsI* and *mrcA* genes. Nevertheless, it should be noted that there might be some other drug efflux pumps besides AcrAB-TolC system on the surface of *H. parasuis*. Overexpression of these efflux pumps might still induce resistance of *H. parasuis* to CEC.

To investigate the antibiotic-resistance reversal effect of MT, the clinical strain C7 was treated with various concentrations of MT (0, 0.125, 0.25, 2, 8, and 16 μg/mL) for predetermined periods of time (1, 4, 7, 10, and 13 h). The concentrations and incubation time were chosen according to the plasma pharmacokinetic (PK) profiles of MT in rat (Jiang et al., 2015) and human (Zhang et al., 2009). When rat was administered orally at a dose of 10 mg/kg, the peak plasma concentration was 1.44 ± 0.14 μg/mL, and the elimination half-life was 7.80 ± 2.25 h. The corresponding PK parameters were 2.38 ± 0.72 μg/mL and 7.80 ± 0.70 h after the volunteers received a single oral dose of MT 400 mg soft gelatin capsule. For clinical strain C7, the concentration and time of exposure to MT were comparable to the plasma PK profiles of MT in rat and human. Although *H. parasuis* was commonly found in the upper respiratory tract of pigs (Little, 1970), the plasma PK profiles of MT could still be used as an alternative standard for evaluating the therapeutic efficacy because of the high tissue permeability of MT (Yang et al., 2010) and the correlation between plasma and tissue PK profile. From a pharmacokinetic/pharmacodynamic perspective, we believed that the antibiotic-resistance reversal effect of MT observed *in vitro* might also be present *in vivo*.

The antibiotic-resistance reversal effect of MT was proved by the 2-to 8-fold decrease in MIC of CEC against clinical strain C7 and significant down-regulated expression of mutated *ftsI* and *mrcA* genes after MT treatment. However, such changes did not appear to be concentration-or time-dependent. It was interesting that prolonged exposure to high concentration of MT (exposure to 16 μg/mL of MT for 13 h) caused the up-regulated expression of mutated *ftsI* gene (Figure 6), which would allow the clinical strain C7 to regain resistance to CEC. We speculated that this may be due to the sensitive perception of non-lethal effect of MT by *H. parasuis*. In fact, the bacterial membrane of clinical strain C7 became wrinkled and concaved after treatment with MT at 16 μg/mL (although much lower than the MIC) for 7 h (Figure 5). Such significant morphological changes might be sufficient for *H. parasuis* to perceive and respond to the non-lethal effect of MT. The mechanism of *H. parasuis* perceiving and responding to the resistance-reversal agent should be further investigated to achieve a significant and long-lasting antibiotic-resistance reversal effect.

## Conclusion

In summary, the mutations in PBP genes (*ftsI* and *mrcA*) could increase the resistance of clinical strain C7 to CEC; MT could reverse the resistance of *H. parasuis* to CEC by inhibiting the mutations in *ftsI* and *mrcA genes*. A further study on the mechanism of *H. parasuis* perceiving and responding to the resistance-reversal agent should be undertaken.

## Data availability statement

The datasets presented in this study can be found in online repositories at the NCBI under accession numbers: 391471, 391472, 391473, 391474.

## Author contributions

JZ: Data curation, Formal analysis, Methodology, Software, Validation, Visualization, Writing – original draft. WY: Data curation, Formal analysis, Methodology, Software, Validation, Visualization, Writing – original draft. HD: Data curation, Methodology, Writing – review & editing. DoL: Data curation, Resources, Software, Writing – original draft. QW: Data curation, Formal analysis, Writing – original draft. LY: Methodology, Writing – review & editing. QK: Methodology, Validation, Writing – original draft. RX: Data curation, Validation, Writing – original draft. DiL: Validation, Visualization, Writing – original draft. RL: Validation, Visualization, Writing – original draft. DY: Supervision, Writing – original draft, Writing – review & editing. BY: Conceptualization, Funding acquisition, Project administration, Supervision, Writing – original draft, Writing – review & editing.
